# Supplementary material for: COVID-19, government measures and hospitality industry performance
Source: PLoS One. 2021 Aug 6;16(8):e0255819. doi: 10.1371/journal.pone.0255819 (PMC8345838; doi:10.1371/journal.pone.0255819)
Supplement: S1 Appendix — (DOCX) [file pone.0255819.s001.docx]

**S1 Appendix. Additional results for Different time windows.**

**TABLE A1 - The Impact of Contact Tracing Measures (h3) on the Hospitality Industry and Industries Closely Related to It**

| Industry | 17/01/2020 | 21/01/2020 | 22/01/2020 |  |
| --- | --- | --- | --- | --- |
|  | **AR_-1_** | **AR_0_** | **AR_+1_** | **CAR[-1,+1]** |
| Food Products | -0.025 | 0.535 | 0.041 | 0.552 |
| Candy & Soda | -0.679 | 1.077 | -0.824 | -0.426 |
| Beer & Liquor | **-2.990^**^** | 0.517 | 1.369 | -1.103 |
| Entertainment | 0.707 | -0.789 | 0.455 | 0.373 |
| Consumer Goods | 0.057 | 0.423 | -0.687 | -0.205 |
| Apparel | -0.872 | -0.438 | -0.327 | -1.637 |
| Personal Services | -1.365^*^ | -0.161 | -0.120 | -1.648 |
| Transportation | -0.767 | **-1.533^*^** | -0.995 | **-3.296^**^** |
| Retail | 0.234 | -0.152 | -0.121 | -0.038 |
| HOSPITALITY | 0.027 | -0.502 | 0.144 | -0.330 |

Notes: The table presents the event study results of the contact tracing (h3) intervention by the U.S. government. The event date is 21/01/2020. AR stands for abnormal returns from the well-known market model by Sharpe (1964), and CAR is the cumulative abnormal returns in a time window that spans t-1 to t+1. Following Brown and Warner (1985) t-statistics for AR and CAR are computed for each industry portfolio. Significant AR and CAR appear in bold, and "***," "**" and "*" denote statistical significance at the 1%, 5% and 10% levels, respectively.

**TABLE A2 - The Impact of international Travel Restrictions (c8) on the Hospitality Industry and Industries Closely Related to It**

| Industry | 31/01/2020 | 03/02/2020 | 04/02/2020 |  |
| --- | --- | --- | --- | --- |
|  | **AR_-1_** | **AR_0_** | **AR_+1_** | **CAR[-1,+1]** |
| Food Products | **-1.325^**^** | -0.244 | -0.434 | **-2.004^**^** |
| Candy & Soda | 0.842 | 0.811 | 1.649 | 3.304 |
| Beer & Liquor | 0.296 | -0.987 | 0.537 | -0.153 |
| Entertainment | -0.858 | -0.174 | 0.819 | -0.213 |
| Consumer Goods | -0.634 | -0.184 | -0.247 | -1.066 |
| Apparel | -1.034 | -0.150 | 0.349 | -0.835 |
| Personal Services | -0.482 | 0.065 | 0.018 | -0.397 |
| Transportation | -0.601 | -0.564 | 0.081 | -1.085 |
| Retail | -0.451 | 0.294 | -1.007 | -1.163 |
| HOSPITALITY | -0.110 | -0.703 | 0.380 | -0.433 |

Notes: The table presents the event study’s results of the *international travel restrictions* (c8) intervention by the U.S. government. The event date is 02/02/2020, which fell on Sunday. Therefore, the effective date was Monday, 03/02/2020. The rest of the notations are as in Table 4.

**TABLE A3 - The Impact of Debt Contract Relief (e2) on the Hospitality Industry and Industries Closely Related to It**

| Industry | 26/02/2020 | 27/02/2020 | 28/02/2020 |  |
| --- | --- | --- | --- | --- |
|  | **AR_-1_** | **AR_0_** | **AR_+1_** | **CAR[-1,+1]** |
| Food Products | **-1.117^*^** | -0.680 | **-1.598^***^** | **-3.396^***^** |
| Candy & Soda | -1.196 | -2.068 | 0.502 | -2.762 |
| Beer & Liquor | -1.463 | 0.017 | -1.559 | -3.005 |
| Entertainment | **-2.941^***^** | 0.617 | 0.173 | **-2.149^*^** |
| Consumer Goods | -0.981 | **1.963^***^** | -0.676 | 0.304 |
| Apparel | -0.573 | **1.710^**^** | 0.468 | 1.604 |
| Personal Services | **-1.817^**^** | 0.471 | 1.139 | -0.206 |
| Transportation | **-1.912^**^** | 1.316 | 0.338 | -0.257 |
| Retail | -1.368 | 1.355 | -0.009 | -0.022 |
| HOSPITALITY | **-3.215^***^** | **-1.279^**^** | -0.864 | **-5.360^***^** |

Notes: The table presents the event study results of the *Debt Contract Relief* (e2) intervention by the U.S. government. The event date was 27/02/2020. The rest of the notations are as in Table 4.

**TABLE A4 - The Impact of Testing Policy (h2) on the Hospitality Industry and Industries Closely Related to It**

| Industry | 27/02/2020 | 28/02/2020 | 02/03/2020 |  |
| --- | --- | --- | --- | --- |
|  | **AR_-1_** | **AR_0_** | **AR_+1_** | **CAR[-1,+1]** |
| Food Products | -0.680 | **-1.598^***^** | **1.471^**^** | -0.807 |
| Candy & Soda | -2.068 | 0.502 | 1.361 | -0.204 |
| Beer & Liquor | 0.017 | -1.559 | **-2.406^*^** | -3.948 |
| Entertainment | 0.617 | 0.173 | **-4.601^***^** | **-3.810^***^** |
| Consumer Goods | **1.963^***^** | -0.676 | **-2.169^***^** | -0.883 |
| Apparel | **1.710^**^** | 0.468 | **-3.556^***^** | -1.378 |
| Personal Services | 0.471 | 1.139 | **-3.071^***^** | -1.460 |
| Transportation | 1.316 | 0.338 | **-4.747^***^** | **-3.092^**^** |
| Retail | 1.355 | -0.009 | **-3.452^***^** | -2.106 |
| HOSPITALITY | **-1.279^**^** | -0.864 | **-2.050^***^** | **-4.195^***^** |

Notes: The table presents the event study results of the *Testing Policy* (h2) intervention by the U.S. government. The event date was 28/02/2020. The rest of the notations are as in Table 4.

**TABLE A5 - The Impact of Canceling Public Events** **(c3) on the Hospitality Industry and Industries Closely Related to It**

| Industry | 28/02/2020 | 02/03/2020 | 03/03/2020 |  |
| --- | --- | --- | --- | --- |
|  | **AR_-1_** | **AR_0_** | **AR_+1_** | **CAR[-1,+1]** |
| Food Products | **-1.598^***^** | **1.471^**^** | 0.175 | 0.048 |
| Candy & Soda | 0.502 | 1.361 | 1.518 | 3.383 |
| Beer & Liquor | -1.559 | **-2.406^*^** | 0.760 | -3.205 |
| Entertainment | 0.173 | **-4.601^***^** | **-1.480^**^** | **-5.908^***^** |
| Consumer Goods | -0.676 | **-2.169^***^** | 0.722 | **-2.123^**^** |
| Apparel | 0.468 | **-3.556^***^** | 0.564 | **-2.523^*^** |
| Personal Services | 1.139 | **-3.071^***^** | 0.543 | -1.388 |
| Transportation | 0.338 | **-4.747^***^** | **1.362^*^** | **-3.045^**^** |
| Retail | -0.009 | **-3.452^***^** | 0.743 | **-2.718^*^** |
| HOSPITALITY | -0.864 | **-2.050^***^** | -0.479 | **-3.394^***^** |

Notes: The table presents the event study results of the *canceling public events* (c3) intervention by the U.S. government. The event date was 01/03/2020 which fell on Sunday. Therefore, the effective date was the Monday 02/03/2020. The rest of the notations are as in Table 4.

**TABLE A6 - The Impact of School Closing (c1) on the Hospitality Industry and Industries Closely Related to It**

| Industry | 04/03/2020 | 05/03/2020 | 06/03/2020 |  |
| --- | --- | --- | --- | --- |
|  | **AR_-1_** | **AR_0_** | **AR_+1_** | **CAR[-1,+1]** |
| Food Products | 0.756 | 0.110 | 0.315 | 1.182 |
| Candy & Soda | 0.673 | -1.411 | 0.682 | -0.056 |
| Beer & Liquor | -1.480 | 0.990 | 0.426 | -0.063 |
| Entertainment | **-2.073^***^** | **-3.694^***^** | **-1.337^*^** | **-7.105^***^** |
| Consumer Goods | -0.240 | -0.730 | 0.638 | -0.331 |
| Apparel | -1.290 | -0.873 | 0.850 | -1.313 |
| Personal Services | -0.948 | -0.771 | -0.807 | **-2.528^**^** |
| Transportation | **-1.791^**^** | **-1.415^*^** | 1.304 | -1.902 |
| Retail | -1.151 | 0.195 | 0.831 | -0.124 |
| HOSPITALITY | **-1.957^***^** | **-3.786^***^** | -0.660 | **-6.404^***^** |

Notes: The table presents the event study results of the *school closing* (c1) intervention by the U.S. government. The event date was 05/03/2020. The rest of the notations are as in Table 4.

**TABLE A7 - The Impact of restrictions on gathering (c4) on the Hospitality Industry and Industries Closely Related to It**

| Industry | 10/03/2020 | 11/03/2020 | 12/03/2020 |  |
| --- | --- | --- | --- | --- |
|  | **AR_-1_** | **AR_0_** | **AR_+1_** | **CAR[-1,+1]** |
| Food Products | 0.152 | 0.242 | **-3.480^***^** | **-3.085^***^** |
| Candy & Soda | 2.448 | -1.313 | -1.504 | -0.370 |
| Beer & Liquor | **-2.357^*^** | -1.503 | **-3.586^**^** | **-7.447^***^** |
| Entertainment | 0.738 | **-5.657^***^** | **-9.644^***^** | **-14.561^***^** |
| Consumer Goods | **-2.274^***^** | **-1.611^***^** | **-4.210^***^** | **-8.096^***^** |
| Apparel | **-3.089^***^** | -0.108 | -1.066 | **-4.263^***^** |
| Personal Services | **-3.287^***^** | **-2.484^***^** | **-3.107^***^** | **-8.879^***^** |
| Transportation | -0.774 | 0.196 | 0.702 | 0.123 |
| Retail | **-3.612^***^** | -1.355 | **-1.679^*^** | **-6.648^***^** |
| HOSPITALITY | -0.086 | **-5.550^***^** | **-4.858^***^** | **-10.490^***^** |

Notes: The table presents the event study results of the *restrictions on gathering* (c4) intervention by the U.S. government. The event date was 11/03/2020. The rest of the notations are as in Table 4.

**TABLE A8- The Impact of Mixed (1) Intervention Stay at Home Requirements (c7), Domestic Travel (c6) and Public Information Campaign (h1) on the Hospitality Industry and Industries Closely Related to It**

| Industry | 13/03/2020 | 16/03/2020 | 17/03/2020 |  |
| --- | --- | --- | --- | --- |
|  | **AR_-1_** | **AR_0_** | **AR_+1_** | **CAR[-1,+1]** |
| Food Products | **0.977^*^** | **-1.053^*^** | **4.658^***^** | **4.581^***^** |
| Candy & Soda | **-4.598^***^** | **-3.836^**^** | **3.474^**^** | **-4.960^*^** |
| Beer & Liquor | 1.824 | 1.381 | 0.459 | 3.665 |
| Entertainment | **2.759^***^** | **-9.954^***^** | **-9.102^***^** | **-16.29^***^** |
| Consumer Goods | **-1.528^**^** | **-2.003^***^** | -0.557 | **-4.089^***^** |
| Apparel | **-5.076^***^** | **-1.915^**^** | **-4.947^***^** | **-11.94^***^** |
| Personal Services | 0.840 | **-4.279^***^** | -0.563 | **-4.002^***^** |
| Transportation | **-2.694^***^** | **1.912^**^** | -0.091 | -0.872 |
| Retail | **-1.953^**^** | 0.029 | **-3.203^***^** | **-5.127^***^** |
| HOSPITALITY | **-1.623^***^** | **-10.29^***^** | **-10.06^***^** | **-21.98^***^** |

Notes: The table presents the event study results of interventions made simultaneously by the U.S. government: *Stay at Home Requirements* (c7), *Domestic Travel* (c6) and *Public Info Campaign* (h1). The event date was 16/03/2020. The rest of the notations are as in Table 4.

**TABLE A9- The Impact of the Closing of Public Transportation (c5) on the Hospitality Industry and Industries Closely Related to It**

| Industry | 16/03/2020 | 17/03/2020 | 18/03/2020 |  |
| --- | --- | --- | --- | --- |
|  | **AR_-1_** | **AR_0_** | **AR_+1_** | **CAR[-1,+1]** |
| Food Products | **-1.053^*^** | **4.658^***^** | **-4.459^***^** | -0.855 |
| Candy & Soda | **-3.836^**^** | **3.474^**^** | **-5.824^***^** | **-6.186^**^** |
| Beer & Liquor | 1.381 | 0.459 | **-3.245^**^** | -1.404 |
| Entertainment | **-9.954^***^** | **-9.102^***^** | **-11.53^***^** | **-30.59^***^** |
| Consumer Goods | **-2.003^***^** | -0.557 | **-4.042^***^** | **-6.603^***^** |
| Apparel | **-1.915^**^** | **-4.947^***^** | **-5.234^***^** | **-12.09^***^** |
| Personal Services | **-4.279^***^** | -0.563 | **-8.130^***^** | **-12.97^***^** |
| Transportation | **1.912^**^** | -0.091 | **-5.162^***^** | **-3.341^**^** |
| Retail | 0.029 | **-3.203^***^** | **-2.688^***^** | **-5.862^***^** |
| HOSPITALITY | **-10.29^***^** | **-10.06^***^** | **-8.358^***^** | **-28.72^***^** |

Notes: The table presents the event study results of the *Public Transportation Closing* (c5) intervention by the U.S. government. The event date was 17/03/2020. The rest of the notations are as in Table 4.

**TABLE A10- The Impact of the Closing of Workplaces (c2) and Aid Packages (e3) on the Hospitality Industry and Industries Closely Related to It**

| Industry | 18/03/2020 | 19/03/2020 | 20/03/2020 |  |
| --- | --- | --- | --- | --- |
|  | **AR_-1_** | **AR_0_** | **AR_+1_** | **CAR[-1,+1]** |
| Food Products | **-4.459^***^** | **2.789^***^** | 0.441 | -1.228 |
| Candy & Soda | **-5.824^***^** | 0.951 | 1.059 | -3.812 |
| Beer & Liquor | **-3.245^**^** | -1.736 | -0.675 | **-5.656^**^** |
| Entertainment | **-11.53^***^** | **13.15^***^** | **7.672^***^** | **9.292^***^** |
| Consumer Goods | **-4.042^***^** | **6.739^***^** | -0.924 | 1.772 |
| Apparel | **-5.234^***^** | **11.38^***^** | -0.998 | **5.147^***^** |
| Personal Services | **-8.130^***^** | **7.307^***^** | 0.890 | 0.066 |
| Transportation | **-5.162^***^** | **2.940^***^** | **3.045^***^** | 0.822 |
| Retail | **-2.688^***^** | **7.400^***^** | 0.720 | **5.432^***^** |
| HOSPITALITY | **-8.358^***^** | **14.08^***^** | -0.131 | **5.593^***^** |

Notes: The table presents the event study results of made simultaneously: *workplaces closing* (c2) and *Aid Package* (e3) interventions by the U.S government. The event date was 19/03/2020. The rest of the notations are as in Table 4.

**TABLE A11- The Impact of Income Support (e1) on the Hospitality Industry and Industries Closely Related to It**

| Industry | 26/03/2020 | 27/03/2020 | 30/03/2020 |  |
| --- | --- | --- | --- | --- |
|  | **AR_-1_** | **AR_0_** | **AR_+1_** | **CAR[-1,+1]** |
| Food Products | **1.228^**^** | -0.651 | **2.296^***^** | **2.872^***^** |
| Candy & Soda | 1.655 | 1.084 | -0.879 | 1.859 |
| Beer & Liquor | 0.777 | **5.627^***^** | -1.688 | **4.716^*^** |
| Entertainment | **-1.430^*^** | **-3.053^***^** | **-4.894^***^** | **-9.379^***^** |
| Consumer Goods | -0.051 | -0.256 | **-2.909^***^** | **-3.217^***^** |
| Apparel | -0.666 | **-1.961^**^** | **-4.245^***^** | **-6.873^***^** |
| Personal Services | 1.035 | -0.283 | **-1.478^**^** | -0.725 |
| Transportation | **-1.744^**^** | **-2.280^***^** | **-4.237^***^** | **-8.262^***^** |
| Retail | **-3.104^***^** | -0.628 | **-4.040^***^** | **-7.772^***^** |
| HOSPITALITY | **1.504^***^** | **-5.170^***^** | **-4.586^***^** | **-8.252^***^** |

Notes: The table presents the event study results of the *Public Transportation Closing* (c5) intervention by the U.S. government. The event date was 27/03/2020. The rest of the notations are as in Table 4.
