# Supplementary material for: COVID-19, government measures and hospitality industry performance
Source: PLoS One. 2021 Aug 6;16(8):e0255819. doi: 10.1371/journal.pone.0255819 (PMC8345838; doi:10.1371/journal.pone.0255819)
Supplement: Attachment — Submitted filename: Response to Reviewers.docx [file pone.0255819.s003.docx]

**Reply to the Editor**

**Manuscript No.: PONE-D-21-07279R2**

**Title: COVID-19, Government Measures and Hospitality Industry Performance**

**PLOS ONE**

We would like to thank you for your constructive comments. We tried our best to answer the points raised and incorporate them into the revised manuscript. We definitely believe that your comments improved the clarity, readability and quality of the paper. They are addressed here in the order they appear in your report followed by our reply.

**Editor comments**

**Thank you for submitting your manuscript to PLOS ONE. After careful consideration, we feel that it has merit but does not fully meet PLOS ONE’s publication criteria as it currently stands. Therefore, we invite you to submit a revised version of the manuscript that addresses the points raised during the review process.**

**The submission improved in a proper manner, but there are required further revisions towards literature review and policy/practical implications.**

Answer: Thanks for your support and positive spirit. In line with your comments, we did our best to address all of the points raised by the reviewers. Please see below our reply to each comment.

**Please include the following items when submitting your revised manuscript:**

- **A rebuttal letter that responds to each point raised by the academic editor and reviewer(s). You should upload this letter as a separate file labeled 'Response to Reviewers'.**
- **A marked-up copy of your manuscript that highlights changes made to the original version. You should upload this as a separate file labeled 'Revised Manuscript with Track Changes'.**
- **An unmarked version of your revised paper without tracked changes. You should upload this as a separate file labeled 'Manuscript'.**

Answer: Following these points:

- We included a rebuttal letter as a separate file, called 'Response to Reviewers'.
- We uploaded a separate marked-up copy of our manuscript labeled 'Revised Manuscript with Track Changes'. The marked-up manuscript uses “track changes” to meet both the technical requirements of the journal and the concerns raised by the referees.
- We uploaded a separate unmarked copy of our manuscript labeled 'Manuscript'.

Finally, we would like to thank you again for your comments and suggestions.

Sincerely,

The authors

**Reply to Referee#1**

**Manuscript No.: PONE-D-21-07279R2**

**Title: COVID-19, Government Measures and Hospitality Industry Performance**

**PLOS ONE**

We would like to thank you for your constructive comments. In the enclosed revised version, we tried our best to answer the points raised and incorporate them into the revised manuscript. Below, we addressed the points in the order they appear in your report followed by our reply.

**1. I appreciate the authors' effort to incorporate changes based on the initial reviews. I do however think that some clarity was lost in effort to broaden the hospitality sector impacts from COVID-19. It became less clear what challenges were brought on by COVID-19 itself and which were directly related to particular governmental interventions.**

Answer: Thank you for raising this important point. In fact, the most common and major challenge during the spread of COVID-19 was uncertainty. As discussed in the introduction section, it stemmed from two sources: the uncertainty created by the pandemic itself and its spread, and the uncertainty that originated in government actions (interventions) and the potential consequences of such actions in the wake of the pandemic. Hence, the government faced the challenge of reducing the uncertainty originating in both sources. Formally, we added the following.

***“****Our main empirical findings documented here show that the major challenge during the spread of COVID-19 was uncertainty. This uncertainty originated in two different, yet related, sources. The first source stemmed from the pandemic itself and the intensified ambiguity about the real consequences for the economy in terms of the time required for economic recovery, the rapidity of the spread of the infection and its lethality. This contention is confirmed using textual analysis of unique data from approximately 3,000 U.S. newspapers.*

*The second source was related to the uncertainty originating in the government interventions themselves. There was a great deal of ambiguity about the economic and non-economic consequences of government interventions. In addition, the public was uncertain about whether the government planned future interventions.*

*In this spirit, empirical studies have shown that increased ambiguity about government policy and spending has direct implications for the steady state of many macroeconomic variables such as debt, the GDP and consumption (e.g., [25]).*

*To summarize, during a crisis like a pandemic, the leading challenge a government faces is reducing both types of uncertainty. Doing so is vital for industries that are sensitive to such uncertainty such as the hospitality sector.* Policy makers might be well-advised to impose measures with detailed transparency about their long-term plans to promote certainty. Ambiguity about current and future government spending (and stimulus packages) creates uncertainty in the stock market [23] and disrupts many macroeconomic variables such as debt, GDP and consumption (e.g., [25]). Our findings can also help policy makers fine-tune their aid policy and help tourism planners prepare better for possible future government interventions during subsequent epidemics such as COVID-19*."*

**2. The main finding seems to be that governments should reduce uncertainty, but I did not walk away from the paper knowing precisely how that might have been done, or could be done in the future, given that there was such a tremendous amount of uncertainty related to a novel virus circulating.**

Answer: Thanks for this comment. To deal with it, we extended the section entitled "Policy implications and recommendations" in which we added concise guidelines and recommendations for reducing uncertainty. The addition below also addresses the point raised in Comment #4. We added the following:

*"**One method that governments can use to reduce uncertainty and increase public confidence is transparency. Since time is crucial in managing a crisis, government policies should be announced publicly as soon as possible. To deal with the uncertainty related to the spread of the virus itself, governments must continuously provide as much information as possible about the evolution of the pandemic. For example, they can use campaigns, create special pandemic or crisis management websites, and provide contacts and information - all aimed at giving the public the maximum sense of control.*

*Regarding the second type of uncertainty, which relates to government policy, governments should be transparent about the types of interventions they plan to use, how they expect to implement them and their planned duration. Governments should publicize their plans for dealing with economic and non-economic issues, indicating how they will affect individuals, investors, employees, employers and business owners. Moreover, since one of the main concerns is loss of revenue and the instability of the economy, the government should be pro-active and set up vehicles such as a national savings fund to remove any ambiguity about the state's financial ability to manage the crisis. In addition, it should announce the availability of income aid, debt payment relief and fiscal spending that is immediate, widespread and generous. In addition, the public should be informed precisely how the government plans to finance this spending (imposing taxes, increasing deficits and debt ceilings, etc.). Transparent policies may mitigate the uncertainty in the short term and allay fears about the economy."*

**3. The authors also included information about the negative effects of the impacts of** **lay-offs and employment instability on hospitality employees in the lit review, but then does not discuss this in the discussion or findings alongside of their results. Instead, the authors directly suggest that cutting costs (does that not mean labor?) is a recommendation to the industry to perform better post pandemic.**

**As I initially suggested, market prices leave out these types of non-market human/environmental impacts. I think expanding your lit review to discuss some of these impacts requires a more thorough discussion alongside of them and this discussion should inform your recommendations.**

**Literature review and discussion.** We apologize for the unclear flow of the literature review. In light of this comment and the points raised by Reviewer #3, we reorganized the "*COVID-19's impact on the hospitality industry*" section and addressed the direct and the indirect implications of the pandemic for the hospitality industry. We added a discussion about lay-offs and employment instability in the empirical findings section, when we discussed the results of interventions related to workplace closures (c2).

*"Table 3 shows that the good news about the assistance package announced on March 18 during the evening overcame the negative news of workplace closures. This result is both interesting and important given that according to the American Hotel and Lodging Association, job losses in the industry are expected to be extensive. In 2020, job loss was projected to be nearly 630,000, while in 2021 job loss were still expected to be around 546,000. Given that about half of hotel markets, representing 72% of hotel rooms in the US, are still in a recession or depression, it seems that providing economic aid might help calm the adverse effect of the expected job losses and the negative impact of imposed closures. Finally, the economic assistance had a major positive impact not only on the hospitality industry but also on several other industries such as entertainment, apparel and retail (CAR = +9.292%, +5.147% and 5.432%, respectively)."*

Moreover, we also added a short discussion in the *"Implications for policy making for the hospitality sector"*:

*"**Our results also underscore the adverse effect that closures have on the performance of the hospitality industry. Therefore, governments should be extremely transparent before enacting such regulations. They should also follow them with economic support to reduce not just the negative effect of the closures, but also the negative effect of the uncertainty about when the restrictions will be lifted.* *An important recommendation derived from our results is providing financial assistance alongside regulations that close workplaces. As our results hint, it is a key condition for providing significant relief from the detrimental impact of labor market closures. Since the labor market is an important component of the soundness and growth of the economy, especially in the hospitality industry, governments should consider mitigating the negative impact of closures with financial measures that support employees, employers and the economy."*

**4. I also think your recommendations could be more specific, particularly, if government interventions somehow directly increased uncertainty, what would uncertainty reduction policies have looked like or could look like in the future.**

Answer: Please see the **second** answer above dealing with the specific recommendations.

**5. Also, it isn't your fault necessarily, its a peril of trying to do timely research in rapidly fluctuated time period, but now we are seeing a rapid recovery in tourism and hospitality, particularly so in domestic hospitality. Can anything be gathered through an understanding of what sub-sectors or industry types are flourishing in this environment? Is it much of the same as the pre-pandemic or has their been any shift?**

Answer: Thanks for this suggestion. It is indeed an interesting point. We added several important statistics dealing with the expected recovery in the hospitality as well as in a related subindustry: the restaurants.

As you point out, the situation is changing rapidly, and therefore, with your permission, we will leave this interesting avenue for future research that deals with the interaction between systematic and idiosyncratic risks and the performance of equities across different industries. The additional recovery statistics appears at the end of the subsection entitled “*Implications for investing”* with a suggestion for a future research agenda:

*"**In the midst of COVID-19 and its economic implications, several opportunities may arise in other related industries, as evident in the unprecedented growth in their equity prices. According to AHLA's report, the hotel occupancy in the U.S. will increase to 52% in 2021, and to 61% in 2022, compared with 66% level in 2018 – 2019 (*[***https://www.ahla.com/sites/default/files/2021_state_of_the_industry_0.pdf***](https://www.ahla.com/sites/default/files/2021_state_of_the_industry_0.pdf)*). In addition, the report projects that U.S. restaurant sales are about to increase to 11% in 2021 to $731.5 billion, but still far behind 2019's $864.3 billion. In this respect, future studies can examine the performance of hospitality and related industries before, during and after the COVID-19."*

Finally, we would like to thank you again for your comments and suggestions.

Sincerely,

The authors

**Reply to Referee#2**

**Manuscript No.: PONE-D-21-07279R2**

**Title: COVID-19, Government Measures and Hospitality Industry Performance**

**PLOS ONE**

We would like to thank you for your constructive comments. In the enclosed revised version, we tried our best to answer the points raised and incorporate them into the revised manuscript. We definitely believe that your comments improved the clarity, readability and quality of the paper. They are addressed here in the order they appear in your report followed by our reply.

**I have reviewed the article and it fullfill all the suggestions sent previously. It is ready for publication.**

Answer: Thanks for your feedback. We deeply appreciate it.

**Reply to Referee#3**

**Manuscript No.: PONE-D-21-07279R2**

**Title: COVID-19, Government Measures and Hospitality Industry Performance: An Event Study Approach**

**PLOS ONE**

We would like to thank you for your constructive comments. In the enclosed revised version, we tried our best to answer the points raised and incorporate them into the revised manuscript. We definitely believe that your comments improved the clarity, readability and quality of the paper. They are addressed here in the order they appear in your report followed by our reply.

1. **What I meant in my comment was (a) specification what do you understand by hospitality industry [and it was done], and (b) correction of the reference to hospitality industry in the whole body of the text [this still needs to be revised]. Please consider the examples of the mix of terms you use through the text (and this is only from a part of the text!)**

**Title: hospitality industry**

**Abstract, line 53 „hospitality and tourism industries”**

**Then, line 55 “tourism and hospitality”**

**87 “hospitality and tourism sectors”**

**93 "tourism and hospitality stocks” but in line 98 “hospitality stock returns”**

**And then in the empirical section: 250 “firms operating in the hospitality industry”**

Answer: We apologize for our misunderstanding. We went over the entire paper and changed our reference to hospitality, accordingly. Thank you for your sharp eyes!

2. **I have also some reservations to the literature review. Although it was expanded (to meet the review comments), still the literature review in the section “COVID-19 impact on the hospitality industry” (line 195 and the following) could be much better.**

**My first suggestion is to remain more concerned on hospitality industry itself (as this is the central point of your work). This section begins with the reference to statistics by WTTC, which is fine, but provides some general information on COVID-19 impact on TRAVEL AND TOURISM sector. Please try to add some statistics & data that refer directly to hospitality industry itself (e.g. https://hoteltechreport.com/news/tourism-industry-statistics#hotels, empirical works or industry reports)**

Answer: Thanks for this comment, and for the excellent source suggested. To address this comment, we extended the previous general information with the addition of a more focused summary about the hospitality industry itself. We repositioned the paragraphs so that the hospitality information appears first. The addition is as follows:

*"The COVID-19 pandemic had an unprecedented negative impact on the hospitality industry. According to a report published by the American Hotel and Lodging Association (https://www.ahla.com/sites/default/files/recessiondepression_0.pdf), the expected US hotels losses are nearly $83.7 ($51.2) billion in room revenue in 2020 (2021), compared with 2019, while job losses in 2020 (2021) are projected to be nearly 630,000 (546,000). In addition, about half of hotel markets, representing 72% of hotel rooms in the US, are still in a recession or depression. These numbers indicate that most of the hotel industry has a long road to recovery, especially when considering that an occupancy rate of 35% or lower makes it impossible for many hotels to stay open. In Fact, individual hotels and major operators are projecting occupancies below 20% (https://hoteltechreport.com/news/tourism-industry-statistics#hotels)."*

3. **My second suggestion is the revision of the empirical works cited in this section. Overall, it is good that on the literature review you provide examples directly tied to hospitality industry (not travel & tourism). However, still, given the extent of the current debate on COVID-19 impacts on hospitality industry, the evidence you provide in literature review seems scarce. Moreover, it is not convincing why these specific works have been revised in the literature review. As a reader, I am missing a clear “story behind”. What I mean more specifically, you refer to selected works, of very different scope, but there is no clear map that could guide us through these exemplary works cited within. You summarize this by stating (line 243): detrimental effects on the hospitality industry. But for me the employees job security (line 203), fake news (line 224) or depressive symptoms of workers (line 236) seem far away from the main problem (stock returns). As your study highlights the context of performance, you should search for the examples of prior works that conform to the performance-related perspective (loss of income, additional costs, overall – what increases the anxieties of investors). The examples of Hao et al. (line 212) or Zheng et al (line 225) are the right direction. Please try to add some more works of this kind, from a business and performance oriented perspective, to make your discussion more substantial. I recommend also to address the works that have revised the impacts of prior pandemic (SARS in 2002-2003 in particular), as these were the first to communicate the possible extent of the pandemic impact on hospitality industry (given the customer outflow and loss of income).**

Answer: Thanks for this insightful point. We would like to note that Reviewer #2 asked us to include these specific studies in our literature review. Nevertheless, and in line with your comment that these papers deviate from the main goal of our paper, we discussed them in a more concise way.

The strategy used in mapping the related literature is as follows. COVID-19 has not only **directly** affected the hospitality industry performance, but also created collateral damage to the entire business environment, which resulted in **indirect** damage to the performance of the hospitality industry. Thus, the papers suggested by Reviewer #2 can be noted as examples of the possible additional indirect forces behind the poor performance and recovery of the hospitality industry.

We added the following to the end of the section, and left the beginning of the section for our main objective:

*"**Importantly, COVID-19 has not only directly affected the performance of the hospitality industry, but also created collateral damage that might indirectly harm it. The literature suggests several possible additional factors behind the poor performance of the hospitality industry that might delay its future recovery. These effects are evident in the labor force (Jung et al. [42]), its mental health (Yan et al. [43]), and the willingness to travel and the spreading of fake news (Alvarez-Risco et al. [44])."*

Second, to address the latter part of your comment, we added several other papers in the spirit of those of Hao et al. (2020) (line 212) and Zheng et al. (2021). We also added several articles dealing with the SARS pandemic in 2003. The following references were added and summarized in the "COVID-19's impact on the hospitality industry" subsection. We hope you agree that our new subsection, which appears after the reference list, addresses your concerns.

1. Chen, M. H., Jang, S. S., & Kim, W. G. The impact of the SARS outbreak on Taiwanese hotel stock performance: an event-study approach. International Journal of Hospitality Management. 2007; 26(1), 200-212.
2. Chien, G. C., & Law, R. The impact of the Severe Acute Respiratory Syndrome on hotels: a case study of Hong Kong. International Journal of Hospitality management. 2003; 22(3), 327-332.‏
3. Clark, J., Mauck, N., & Pruitt, S. W. The Financial Impact of COVID-19: Evidence from an Event Study of Global Hospitality Firms. Research in International Business and Finance. 2021; 101452.‏
4. Crespí-Cladera, R., Martín-Oliver, A., & Pascual-Fuster, B. Financial distress in the hospitality industry during the Covid-19 disaster. Tourism Management. 2021; 85, 104301.

‏

5. Hao, F., Xiao, Q. and Chon, K. COVID-19 and China’s Hotel Industry: Impacts, a Disaster Management Framework, and Post-Pandemic Agenda. International Journal of Hospitality Management. 2020; Vol. 90, September 102636.
6. Henderson, J. C., & Ng, A. Responding to crisis: severe acute respiratory syndrome (SARS) and hotels in Singapore. International Journal of Tourism Research. 2004; 6(6), 411-419.‏
7. Kim, S. S., Chun, H., & Lee, H. The effects of SARS on the Korean hotel industry and measures to overcome the crisis: A case study of six Korean five-star hotels. Asia Pacific Journal of Tourism Research. 2005; 10(4), 369-377.
8. Lee, C-C, Lee, C-C, Wu, Y. The impact of COVID-19 pandemic on hospitality stock returns in China. Int J Fin Econ. 2021; 1– 14. <https://doi.org/10.1002/ijfe.2508>
9. Rodríguez-Antón, J. M., & Alonso-Almeida, M. D. M. COVID-19 Impacts and Recovery Strategies: The Case of the Hospitality Industry in Spain. Sustainability. 2020; 12(20), 8599.‏
10. Tew, P. J., Lu, Z., Tolomiczenko, G., & Gellatly, J. SARS: lessons in strategic planning for hoteliers and destination marketers. International Journal of Contemporary Hospitality Management.‏ 2008; Apr 18; 20(3): 332–346.
11. Zheng, D. Luo, Q. and Ritchie, B.W. Afraid to travel after COVID-19? Self-protection, coping and resilience against pandemic ‘travel fear’. 2021; Tourism Management 83, 104261.

## COVID-19's impact on the hospitality industry

*"**The COVID-19 pandemic had an unprecedented negative impact on the hospitality industry. According to a report published by the American Hotel and Lodging Association (https://www.ahla.com/sites/default/files/recessiondepression_0.pdf), the expected US hotels losses are nearly $83.7 ($51.2) billion in room revenue in 2020 (2021), compared with 2019, while job losses in 2020 (2021) are projected to be nearly 630,000 (546,000). In addition, about half of hotel markets, representing 72% of hotel rooms in the US, are still in a recession or depression. These numbers indicate that most of the hotel industry has a long road to recovery, especially when considering that an occupancy rate of 35% or lower makes it impossible for many hotels to stay open. In Fact, individual hotels and major operators are projecting occupancies below 20% (https://hoteltechreport.com/news/tourism-industry-statistics#hotels).*

*Similarly, the general state of the travel and tourism industry is also under a great threat. According to the Economic Impact Report by the World Travel and Tourism Council [30], prior to the pandemic, the travel and tourism sectors, both directly and indirectly, accounted for 1 in 4 of all new jobs created around the world, 10.6% of all jobs (334 million), and 10.4% of global GDP (US$9.2 trillion). In 2020, 62 million jobs were lost, representing a drop of 18.5% (62/334). The threat of job losses is continuing as many jobs are currently supported by government retention schemes and reduced hours, which without a full recovery in this sector, could be lost.*

*A careful mapping of the literature shows that several papers have reported supporting evidence for COVID-19’s adverse effect on the performance of the hospitality sector. Hao et al. [31] reviewed the overall impact of the pandemic on China’s hospitality industry - the country where the health crisis began. The industry witnessed a sharp decline in hotel occupancy rates and a loss of over US$9 billion in revenue. About 74% of the hotels in China were closed in January and February 2020 for an average period of 27 days. Furthermore, from January 14 to 28, the occupancy of the hotels dropped from around 70% to 8% and remained under 10% in the following 28 days. As a result, the hotel and tourism industry reduced their number of employees, leading to a significant drop in cash flow and revenue. Zheng et al. [32] studied the phenomenon of “travel fear” in China. They reported that perceptions about the severity of the threat and the susceptibility to it can cause “travel fear,” which leads to protective behaviors with regard to travel decisions. Furthermore, “travel fear” can evoke different strategies that increase people’s psychological resilience and adoption of cautious travel behaviors. Villace-Molinero et al. [33] explored perceptions about travel risks during the pandemic and proposed measures to improve traveler confidence based on the issue-attention cycle. Based on a survey conducted in 46 countries and a qualitative study in which 28 international hospitality experts were interviewed, the authors concluded that in a pandemic scenario, confidence in communications from the local government about personal safety and security are the main factors people consider when making travel decisions.*

*Lee et al. [34] tested the impact of COVID-19 on hospitality stock returns in China. They argue that the increasing uncertainty about the COVID-19 outbreak has made the Chinese stock market more turbulent and less predictable. Using a structural vector auto regression (SVAR) framework, they examined the link between the COVID-19 outbreak, macroeconomic fluctuations and hospitality stock returns in China. Their results hint that macroeconomic fluctuations and hospitality stock returns are significantly affected by shocks from the COVID-19 outbreak. Crespí-Cladera et al. [35] used a stress methodology to estimate the potential performance vulnerability for Spanish hospitality firms. They demonstrated that almost 25% of their sample firms are exposed to financial distress when operational income decreases 60%. They also found that the majority of such firms are generally small ones, which would also suffer from solvency problems. When hospitality firms’ revenues drop 80%, the predictions show that 32% of firms would be in financial distress. Rodríguez-Antón and Alonso-Almeida [36] reported that the performance of the hospitality industry in Spain has been severely damaged as a result of COVID-19. More specifically, they noted that in the first seven months in 2020, the total hotel overnight stays in Spain declined from 184.7 million in 2019 to nearly 46.4 million. In addition, the pandemic reduced the number of new hotel openings (−22.02%) and the number of employees hired (−30.94%) in March 2020. Finally, a recent study of Clark et al. [37] documented the negative impact of COVID-19 on the stock performance of hospitality firms. They estimated negative mean cumulative abnormal returns of −17.54% for 54 publicly traded hospitality firms from 23 different countries. Restricting their sample to the US or Japan yielded negative cumulative abnormal returns of −29.67% and −10.68%, respectively.*

*Other studies have examined the impact of previous pandemics, such as the severe acute respiratory syndrome (SARS), on the performance of the hospitality industry. Chien and Law [38] showed that the outbreak of SARS in March 2003 had a strong negative impact on the hotel business in Hong Kong. The occupancy rates of many hotels in Hong Kong fell to 10% or less in March and April 2003, which normally is the peak season. Similarly, Hendersom and Ng [39] reported statistics from the Singapore Tourism Board confirming the severity of the impact of SARS on the hospitality sector there. According to the report, the average hotel occupancy rate for the second quarter of 2003 was 21%, compared with 74.5% for the previous year, and average room rates dropped by 18.8%. In addition to this report, they also surveyed hotels in Singapore to estimate the economic loss resulting from SARS. They noted that in their own surveyed hotels the average occupancy rates were also relatively low, in the range of 27.7% to 42.3%.*

*Kim et al. [40] tested the effect of SARS on the Korean hotel industry. They examined six Korean hotels and reported that the occupancy rate dropped nearly 14% from February to July 2003. They argued that the reason seemed to be that inbound tourists saw Korea as an unsafe tourism destination within the territory of the SARS-affected Asian Pacific zone. Revenue per available room (RevPAR) during the three months from April to June was 215,849 won (US$180) in 2002, whereas it was 115,676 won (US$96) in 2003, a 100,173 won (US$83) difference. Finally, there was a 16% drop in profit margins from April through June in 2003 compared to the same period in 2002. The average rate per room also declined by 19% as hotels attempted to cope with the sharp decline in demand. Tew et al. [41] used a questionnaire designed to investigate, among other points, the impact of SARS on hotel performance in Canada. Respondents were asked to assess the impact that the SARS crisis had on their hotel's performance. Over 82% of respondents reported that SARS had an extremely negative or very negative impact on their hotel's performance. In addition, Tew et al. (2008) also reported that the Niagara Falls region experienced a loss of over 122,000 room nights in the second quarter of 2003. This loss translated into a loss of $19 million in room revenue. Finally, Chen et al. [17] examined the effect of the SARS epidemic on Taiwanese hotel stock price movements using an event-study approach. They showed that for seven publicly traded hotel companies there were steep declines in earnings and stock prices during the SARS outbreak period. More specifically, they reported that in April 2003 hotel companies experienced drops in earnings in the range of −49.81% to −11.14%. They also showed that these drops worsened significantly when extending the period examined for two months (April – June, 2003), with reductions varying from −76.89% to −20.00%. Finally, they calculated the cumulative abnormal returns of stocks in this sector during 10 and 20-day windows from the day of the SARS outbreak. The negative returns they found were also robust using different types of models to estimate the abnormal returns.*

*Importantly, COVID-19 has not only directly affected the hospitality industry performance, but also created collateral damage that might indirectly harm it. The literature suggests several possible additional factors behind the poor performance of the hospitality industry that might delay its future recovery. These effects are evident in the labor force (Jung et al. [42]), its mental health (Yan et al. [43]), and the willingness to travel and the spreading of fake news (Alvarez-Risco et al. [44]).*

*To summarize, these studies show that in addition to the negative effect that government interventions usually have on financial markets, COVID-19 also had various detrimental effects on the hospitality industry. Therefore, combining these two pieces of evidence, we might expect that the impact of government interventions on the hospitality industry would also be negative."*

Finally, we would like to thank you again for your comments and suggestions.

Sincerely,

The authors
